# The Effects of DNA Extraction Kits and Primers on Prokaryotic and Eukaryotic Microbial Community in Freshwater Sediments

**DOI:** 10.3390/microorganisms10061213

**Published:** 2022-06-14

**Authors:** Zihan Shi, Qiaoyi Kong, Xinghao Li, Wenxin Xu, Chengzhi Mao, Yunfeng Wang, Weibo Song, Jie Huang

**Affiliations:** 1 Institute of Evolution & Marine Biodiversity, College of Fisheries, Ocean University of China, Qingdao 266003, China; zihanshi0909@163.com (Z.S.); yunfengwang1@126.com (Y.W.); wsong@ouc.edu.cn (W.S.); 2 Donghu Experimental Station of Lake Ecosystems, Key Laboratory of Aquatic Biodiversity and Conservation of Chinese Academy of Sciences, Institute of Hydrobiology, Chinese Academy of Sciences, Wuhan 430072, China; kongqiaoyi@ihb.ac.cn (Q.K.); xuwenxinoo@163.com (W.X.); maochengzhi88@163.com (C.M.); 3 Key Laboratory of Regional Development and Environmental Response, Hubei Engineering Research Center for Rural Drinking Water Security, Hubei University, Wuhan 430062, China; li_xhao@163.com

**Keywords:** DNA extraction, primer, freshwater sediment, microbial community

## Abstract

DNA based sequencing technology has revolutionized the field of microbial ecology and environmental studies. However, biases can be introduced at all experimental steps and, thus, affect the interpretation of microbial community. So far, previous studies on the biases introduced from the key steps of DNA extraction and primer sets mainly focused on the bacterial communities in soil or sediment samples, while little is known about the effect on the eukaryotic microbial communities. Here, we studied the effects of three different DNA extraction kits on both prokaryotic and micro-eukaryotic communities by 16S and 18S rRNA gene amplicon sequencing, and further disentangled the influence of primer choice on the micro-eukaryotic communities. Our results showed that the FastDNA SPIN Kit for Soil and DNeasy PowerSoil Kit produced much higher DNA yield with good reproducibility, and observed more eukaryotic OTUs compared to the MinkaGene DNA extraction kit, but all three kits exhibited comparable ability in recovering bacterial alpha diversity. Of the two primer sets, both targeting the V4 region of the 18S rRNA gene, the TAR primer set detected higher number of unique OTUs than the EK primer set, while the EK primer set resulted in longer amplicons and better reproducibility between replicates. Based on our findings, we recommend using the DNeasy PowerSoil Kit with the EK primer set to capture the abundant micro-eukaryotic taxa from freshwater sediment samples. If a more complete picture of the eukaryotic microbial community is desired, the TAR primer set in combination with the FastDNA SPIN Kit is more efficient in this study.

## 1. Introduction

High-throughput sequencing technology is widely adopted for profiling the structure and diversity of microbial communities [1,2,3,4,5,6]. However, the results in different studies are not consistent and comparable as all processing steps from sampling to data analysis may introduce bias [7,8,9,10]. In particular, it has been pointed that a major source of variation between studies may come from DNA extraction and primer selection [11]. The optimal protocol of DNA extraction should be effective to extract DNA from various cell types; otherwise, DNA of microorganisms that are difficult to lyse may not be detected in the sequencing data [12]. Ideally, the primer sets should successfully amplify all groups of the target organisms, while the reality is that different primer sets result in substantial discrepant species recovery [13]. Thus, any specific combinations of available PCR primers and DNA extraction protocols are not 100% efficient to recover the total microbial diversity [13,14].

It is highly recommended that different DNA extraction methods should be compared to find the optimal protocol for specific samples [15]. Various DNA extraction protocols were recommended as a variety of sample types and different extraction kits are compared [7,16,17]. It has been proposed that DNA extraction protocols involved mechanical lysis or bead-beating step could improve the efficiency of DNA extraction [7,18]. Specifically, DNA extracted from activated sludge samples or a mock community of human microbiome samples using kits with the bead-beating step both produced higher DNA yields and higher abundant operational taxonomic units (OTUs) comparing with kits without the mechanical step [7,19]. Furthermore, some Gram-positive bacteria were clearly underestimated, although protocols with mechanical lysis was used, which may be due to the various bead-beating time and efficiency of cell lysis among different kits [16]. DNA extraction efficiency can also be affected by the biomass and diversity of the microbial community in different depths of marine sediment. For example, two widely used DNA extraction kits (FastDNA spin kit for soil and DNeasy PowerSoil kit, referred as the FS kit, and the PS kit hereinafter, respectively) exhibit the same ability to extract DNA from sub-seafloor samples, while higher DNA yields are obtained using the FS kit from surface near-seafloor sediment [17]. For porcine gastrointestinal tract and activated sludge samples, the FS kit is uniformly recommended as it can produce the purest DNA with higher DNA yield and be more efficient in detection of Gram-positive bacteria [7,16]. By contrast, some results showed that the PS kit is the most effective method to extract microbial DNA from human stool samples [20]. Another commercial kit, the NucleoSpin Soil kit performed best in quantification of bacteria from pig manure samples [21]. A recent study has shown that the rare plankton subcommunities from the freshwater reservoir are far more affected by DNA extraction kits than the abundant plankton. The FS kit outperforms the other extraction methods in DNA quality and yield, which results in revealing higher bacterioplankton diversity while the DNeasy Blood & Tissue Kit and QIAamp DNA Mini Kit exhibits better reproducibility [22]. As shown above, there are increasing studies of evaluating the impact of various DNA extraction methods on bacterial community profiles from different source of samples. However, a comprehensive assessment of freshwater sediment extraction methods, especially the data pertaining to micro-eukaryotic community, is still lacking.

It should also be noted that primers targeting different hypervariable regions of the rRNA genes do not recover consistent adequate representation of biodiversity [23,24]. For the same variable region, there remain different primer selections. Even a single primer-template mismatch at the 3′ end of primer or the introduction of one single degenerate base may inhibit the PCR efficiency and lead to discrepancy in microbial community analysis. Thus, it is necessary to estimate the universality and efficiency of different primer sets for various environment samples in microbial biodiversity studies [24,25].

To address the above-mentioned issues, three commercial DNA extraction kits were compared to extract DNA from freshwater sediment in terms of DNA yields, quality and reproducibility. Both bacterial and eukaryotic community profiles were investigated by Illumina Miseq sequencing of 16S and 18S rRNA gene amplicons. To explore the effect of primer choice, the consequences of two widely used primer sets targeting the V4 region of the 18S rRNA gene were also assessed.

## 2. Materials and Methods

### 2.1. Sample Collection and Processing

Freshwater sediment was collected using a sediment corer from the second largest urban lake of China, Lake Donghu (30°33′08″ N, 114°21′57.8376″ E) on 15 September 2018. The sample was transported to the lab immediately (within 10 min). The top 5 cm of sediment was mixed and homogenized, and divided into 15 subsamples with 0.5 g for each aliquot.

### 2.2. DNA Extraction, PCR and Sequencing

DNA extraction of sediment samples was performed using three commercial kits according to the manufacturer’s instructions. Five replicates for each of the following kits were evaluated: DNeasy PowerSoil Kit (Qiagen, Hilden, Germany), FastDNA SPIN Kit for Soil (MP Biomedicals, Solon, OH, USA), and MinkaGene Soil DNA Kit (Magigene Biotechnology, Shenzhen, China), which were referred as PS, FS, and MG, respectively. In all the three protocols, DNA was eluted using 100 μL elution buffer. DNA concentration and purity was measured using NanoDrop 2000 spectrophotometer (Thermo Fisher Scientific, Waltham, MA, USA). The primers used to amplify the V4 hypervariable region of the 16S rRNA gene were 515F (5′-GTGCCAGCMGCCGCGGTAA-3′) and 806R (5′-GGACTACHVGGGTWTCTAAT-3′) [26,27]. Two widely used primer sets were selected to amplify the V4 region of the 18S rRNA gene: TAReukV4F (5′-CCAGCASCYGCGGTAATTCC-3′) and RevR (5′-ACTTTCGTTCTTGATYRA-3′), and EK-565F (5′-GCAGTTAAAAAGCTCGTAGT-3′) and EK-1134R (5′-TTAAGTTTCAGCCTTGCG-3′) [28,29]. PCR reaction was performed in a total volume of 20 μL containing 4 μL of 5× FastPfu Buffer, 2 μL of 2.5 mM dNTPs, 0.8 μL of each primer (5 μM), 0.4 μL of FastPfu Polymerase, 0.2 μL BSA, and 10 ng of template DNA. In PCR of the 16S rRNA gene, the following cycling conditions were used: initial denaturation at 95 °C for 3 min, followed by 27 cycles of 95 °C 30 s, 55 °C for 30 s, 72 °C for 45 s, and elongation at 72 °C for 10 min. PCR of the 18S rRNA gene using the EK-565F/1134R primer set were performed under the same PCR conditions as above described, except that 35 cycles were used. PCR parameters of the 18S with the primer set TAReukV4F/RevR were as follows: initial activation at 95 °C for 5 min, followed by 30 cycles of 94 °C 30 s, 47/49 °C for 45 s, 72 °C for 1 min, and a final extension at 72 °C for 5 min. To minimize PCR bias, PCR amplification was performed in triplicates. Purified PCR products were pooled and paired-end sequencing (2 × 300 bp) was conducted on an Illumina MiSeq platform (Illumina, San Diego, CA, USA) as previously described [30].

### 2.3. Sequence Data Processing and Taxonomic Assignment

Raw data were processed following the standard procedures of Majorbio (Shanghai, China) as described in Li et al. [30]. Briefly, paired-end reads were quality-filtered by Trimmomatic and merged by FLASH [31,32]. UCHIME was used to identify and remove chimeric sequences [33]. Operational taxonomic units (OTUs) were clustered using UPARSE version 7.1 (http://drive5.com/uparse/ (accessed on 27 February 2019)) according to 97% similarity [34]. Taxonomic classification of OTUs were performed using the Silva (release 138) 16S rRNA database and the PR2 18S rRNA gene database, respectively. After filtering the unclassified domain OTUs and singletons, metazoa, and fungi sequences, all analyses were performed on randomly rarefied subsamples (n = 33,726 sequences for 16S; n = 21,079 sequences for 18S).

### 2.4. Statistical Analysis

All analyses were conducted on the cloud platform of Majorbio (https://cloud.majorbio.com (accessed on 10 August 2021)), if not stated otherwise. To evaluate the influence of DNA extraction kits on DNA yields and quality (i.e., A260/A280 and A260/A230), one-way ANOVA was employed with Tukey–Kramer post-hoc test for multiple pairwise comparisons using PAST. The significant difference of alpha diversity was determined based on the nonparametric Kruskal–Wallis test. Non-metric multidimensional scaling (NMDS) ordination plots were conducted based on the Bray–Curtis distances and unweighted UniFrac phylogenetic distances. Analysis of similarities (ANOMIS) was performed based on Bray–Curtis distances to detect the significance of variation in community structures.

## 3. Results

### 3.1. Effects of DNA Extraction Kits in DNA Yield

**Figure 1:**
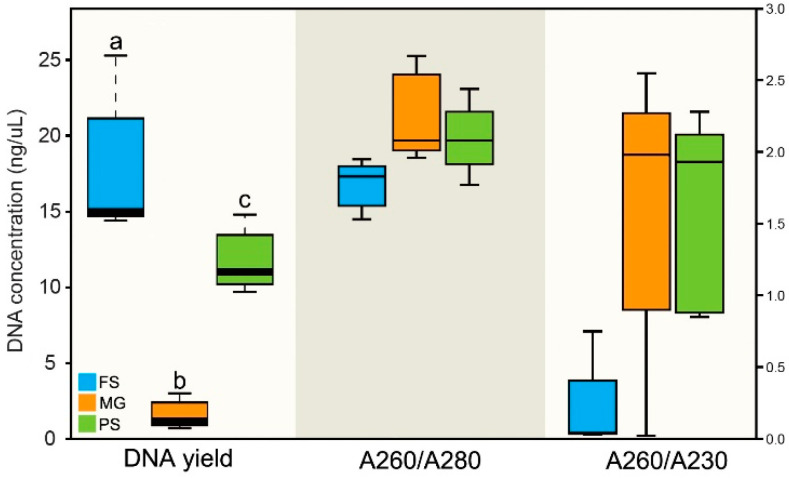
Comparison of DNA yields and quality between three extraction kits. DNA was eluted in a volume of 100 μL for all three kits and qualified by the ratios of A260/A280 and A260/A230. Median values are indicated by the solid line within each box, and the box extends to upper and lower quartile values. Different letters above the box indicate they are statistically different (*p* < 0.05/0.01).

The variance in DNA yields between three different kits were evaluated based on the DNA concentration. As shown in Figure 1, the DNA concentrations varied significantly between samples extracted by different kits (One-way ANOVA, *p* < 0.05/0.01). As shown in Table 1, the MG kit produced the lowest average DNA concentration (mean ± SEM; 1.56 ± 0.18 ng/μL; n = 5), which was statistically lower than that obtained using the PS kit (11.66 ± 0.39 ng/μL; n = 5) and the FS kit (17.34 ± 0.91 ng/μL; n = 5).

Five aliquots of samples were extracted by each DNA extraction kit to evaluate the reproducibility of different extraction methods. As indicated by coefficients of variation (CV) between replicates, the PS kit showed the best reproducibility for DNA yield (PS = 15%, FS = 24%, MG = 51%). For DNA purity, average A260/A280 of the MG kit (mean ± SEM; 2.24 ± 0.06) is higher than the PS (2.09 ± 0.05) and FS (1.78 ± 0.03) kit. Average A260/A230 of the FS kit (0.18 ± 0.06) is significantly lower than the other two methods (PS: 1.59 ± 0.13; MG: 1.66 ± 0.19), indicating that the FS kit had much more residual carryover.

### 3.2. Effects of DNA Extraction Kits on Prokaryotic Microbial Community

**Figure 2:**
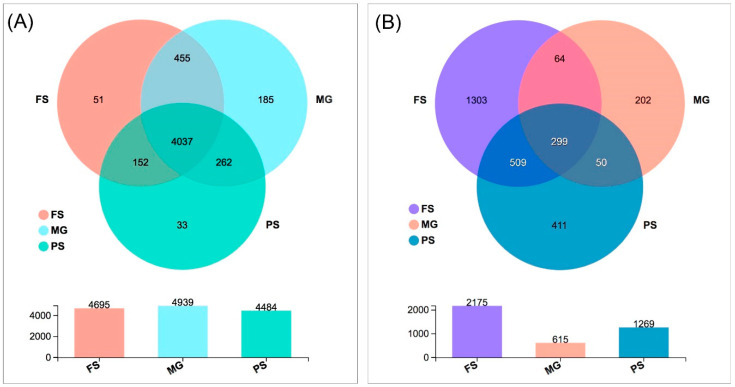
Venn diagram showing the number of shared and unique bacterial (**A**) and eukaryotic (**B**) OTUs among the samples extracted using the FS, MG, and PS kit.

**Figure 3:**
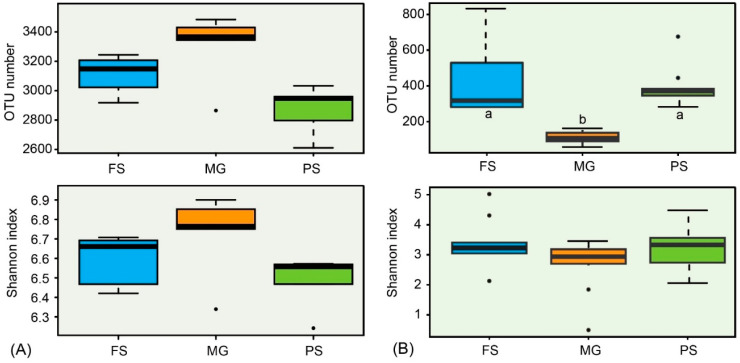
Alpha diversity metrics of prokaryotes (**A**) and microbial eukaryotes (**B**) from samples extracted using the FS, MG, and PS kits. Species richness and diversity were estimated based on the OTU numbers and Shannon index for each method. Different letters above/below the box indicate they are statistically different (*p* < 0.001).

We assessed the impacts of different extraction kits on prokaryotic diversity and community composition. A total of 505,890 prokaryotic reads were obtained after filtering and rarefication, which were clustered into 5175 OTUs for all samples. There were 4484 OTUs, 4695 OTUs, and 4939 OTUs in total for all five replicate samples extracted by the PS, FS, and MG kit, respectively (Figure 2A). The number of observed prokaryotic OTUs were not significantly different between extraction methods, although samples extracted using the MG kit had a higher number of OTUs (3378–4321) than that in other samples (3177–3920 in FS kit and 3005–3668 in PS kit, Figure 3A). Likewise, the Shannon diversity was also statistically indistinguishable between any of the three kits based on Kruskal–Wallis test (Figure 3A). The majority of OTUs (4037) were detected by all three extraction methods, which account for more than 98% of the total bacteria abundance in each sample (Table S1). A small number of unique OTUs with low relative abundances (less than 1%) were also detected by each kit (Figure 2A, Table S1). However, the relative abundance variations of shared taxa are observed between kits. More than one third of the shared taxa at the genus level are differentially abundant between two extraction kits. Among them, the relative abundances of four taxa (g_Clostridium_sensu_stricto_1, g_Anaeromyxobacter, g_norank_o_Syntrophobacterales, g_unclassified_o_Polyangiales) are significantly different between any two kits (Table S2).

**Figure 4:**
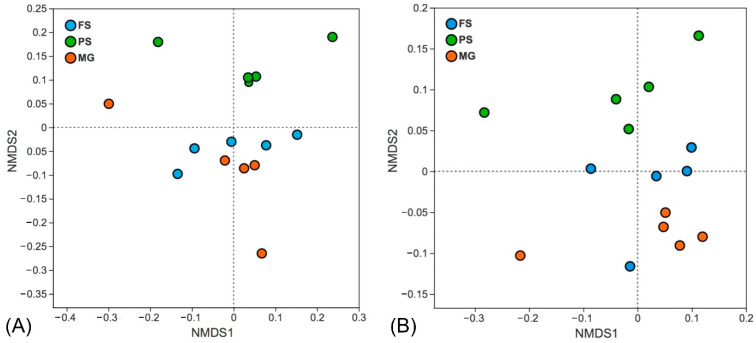
Nonmetric multidimensional scaling ordination of prokaryotic samples extracted by the FS, PS and MG kits based on Bray–Curtis distances (**A**) and unweighted UniFrac phylogenetic distances (**B**).

The bacterial community composition at the phylum level of each sample is shown in Figure S1. The most abundant phyla (>5% in reads abundance) detected by all three extraction kits include Proteobacteria, Chloroflexi, Crenarchaeota, Desulfobacterota, Sva0485, Halobacterota, Acidobacteriota, Bacteroidota, and Thermoplasmatota. To reveal differences in bacterial community composition of samples extracted using different kits, both Bray–Curtis and unweighted UniFrac distances were compared and depicted in Figure 4. We detected a significant difference of the bacterial communities between the DNA extraction kits (ANOSIM, *p* = 0.001). Similar trends were also observed in the NMDS ordination plot, whereas the recovered microbial communities tend to form clusters according to the extraction kits (Figure 4).

### 3.3. Effects of DNA Extraction Kits and Primer Sets on Eukaryotic Microbial Community

After quality control and rarefication, a total of 632,370 eukaryotic sequences that clustered into 2838 OTUs were used in subsequent analyses. As shown in Figure 2B, a total of 2175, 1269, and 615 OTUs were observed for 5 replicate samples extracted by the FS, PS, and MG kit using two primer sets, respectively. Only 299 OTUs are shared by all three extraction methods, accounting for more than 81% of the total reads in all samples. The number of the total detected OTUs by two primer sets were not significantly different between the FS and PS kit samples while the MG kit obtained much lower OTUs than the other two kits. Similarly, a large difference in the number of observed OTUs was also found between primer choice. The EK primer set produced much fewer OTUs (1046) than the TAR primer set (2589). Specifically, the FS kit combined with the TAR primer set detected the highest number of OTUs (1933), followed by the PS kit using the same primers (1118). The lowest OTUs (355) was also observed in samples using this primer set when the extraction kit changed to MG. The number of OTUs detected by another EK primer set using the PS and FS kits was much lower (677–725) than that obtained using the TAR primer set (1118–1933), while the two primer sets detected equivalent OTUs in samples extracted using the MG kit (355 vs. 365, Figure S2).

**Figure 5:**
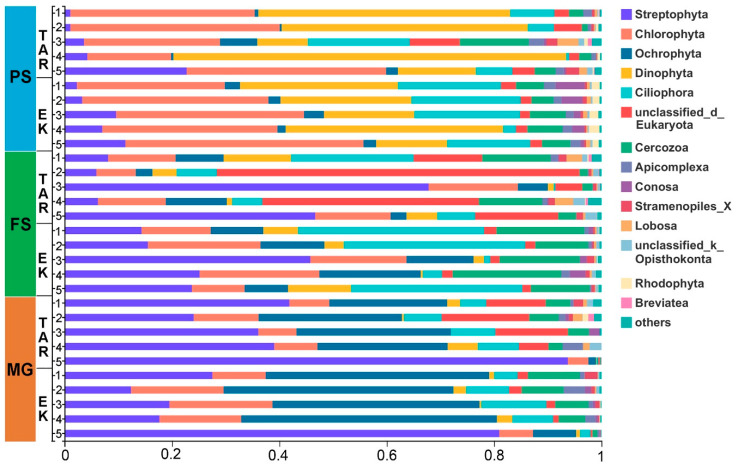
Relative abundances of eukaryotic communities at phylum level detected by two primer sets in samples extracted using the PS, FS, and MG kit.

**Figure 6:**
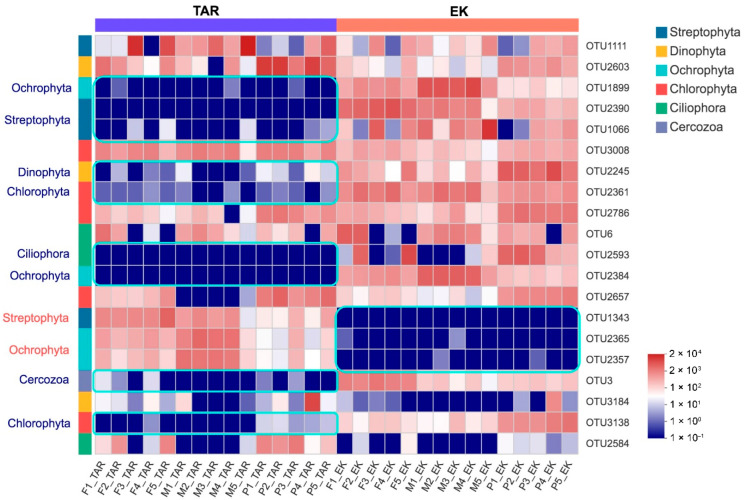
Heatmap of the 20 most abundant OTUs. Each column represents a sample and is grouped by different primer sets. The rows list the 20 most abundant phyla across the samples. Each OTU is assigned with its phylum. OTU1899 and OTU2365 are both assigned in the genus *Stephanodiscus*; OTU2384 and OTU2357 are assigned in the genus *Aulacoseira*.

**Figure 7:**
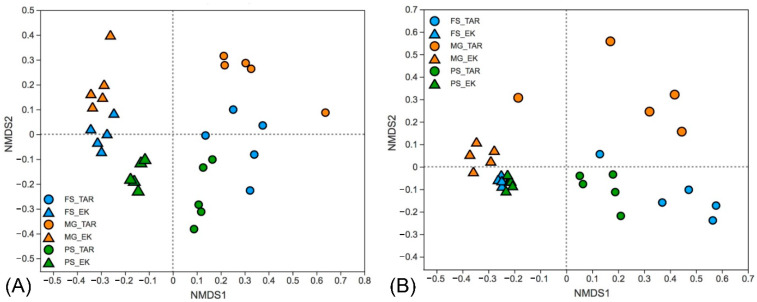
Nonmetric multidimensional scaling ordination of eukaryotic communities detected by two primer sets using the FS, PS and MG kits based on Bray–Curtis distances (**A**) and unweighted UniFrac phylogenetic distances (**B**).

The relative abundance of eukaryotic communities at phylum level varied between extraction methods, the majority of sequences in samples extracted by the PS kit belong to Chlorophyta, Dinophyta, and Ciliophora, while more Streptophyta and Cercozoa sequences was detected by the FS kit. In contrast, the MG kit outperforms the other two kits in detecting Ochrophyta and Streptophyta taxa (Figure 5). Moreover, the varying abundances of the 20 most abundant OTUs were observed between the primer sets, which are distributed in the phyla Ochrophyta, Streptophyta, Chlorophyta, Dinophyta, Ciliophora, and Cercozoa. It is noteworthy that the relative abundances of different OTUs belong to the same genus (i.e., *Stephanodiscus*, *Aulacoseira*) can vary greatly between primer sets (Figure 6). In addition, the non-metric multidimensional scaling ordination plots based on Bray–Curtis dissimilarity and unweighted UniFrac distances both revealed a stark separation in the eukaryotic community profiles between the primer sets (Figure 7, *p* = 0.001). A clear distinction of the three extraction kits was also observed on the NMDS plot based on the Bray–Curtis distance. The EK primer set demonstrated better reproducibility as all replicates clustered more closely whereas the samples detected by the TAR primer set are scattered around, especially when their phylogenetic relatedness (unweighted Unifrac) were considered (Figure 7).

## 4. Discussion

DNA extraction methods and primer sets are known sources of variation in microbial community analysis [11,35,36]. Numerous studies have mainly focused on whether different DNA extraction procedures can affect the bacterial community from a variety of samples, such as human fecal samples [37,38], animal feces [16,21,39], drinking water [40], soils, and marine sediment [17,41]. However, our knowledge about the influence of DNA extraction on microbial community of both bacteria and micro-eukaryotes is very limited [22,42]. In particular, no comprehensive studies have been conducted in freshwater sediment.

In this study, we compared the performance of three commonly used DNA extraction kits with mechanical lysis, which is necessary for DNA extraction from organic-rich sediment samples. Our results demonstrated that the FS kit yielded the highest amounts of DNA which also outperforms other kits in bacterial DNA extraction from different soil samples [43,44]. High amount of DNA recovery is probably attributed to the glass beads of varying sizes and its unique binding matrix of the FS kit [7]. Unexpectedly, although the DNA yields obtained from the FS, PS, MG kits varied remarkably, the number of bacterial OTUs were not significantly different (Figure 3A). Compared with the FS and PS kits, the MG kit produced the lowest amount of DNA, but revealed the highest alpha diversity of bacteria. In addition, even the low-quality DNA could also reveal a fair profile of bacterial community. For example, the FS kit extracted DNA with the lowest ratio of A260/A230, but a reasonable number of OTUs was also observed. Thus, low-quantity or low-quality DNA does not necessarily affect the detection efficiency of bacteria, which has also been found in previous studies [7,44]. However, the MG kit underestimated the eukaryotic microbial diversity as the samples extracted with this kit had a significantly lower number of eukaryotic OTUs (*p* < 0.001, Figure 3B). This suggests that the MG kit may be more efficient in cell lysis of bacterial cell than eukaryotic microorganisms. But the large number of eukaryotic OTUs observed by the FS and PS kits were in low abundance as all of the 20 most abundant OTUs were observed regardless of extraction method (Figure 6), which was also the case for detection of microbial communities in drinking water samples [40]. Similarly, a recent study in freshwater reservoir found that rare plankton subcommunities are far more affected by DNA extraction kits than abundant plankton [22].

In terms of the reproducibility, the MG kit produced a lower DNA yield and exhibited a higher degree of variability between replicates, as indicated by the higher coefficients of variation of DNA yield and the relatively dissimilar pattern from the NMDS plot (Figure 7). This result highlights the importance of the DNA extraction efficiency. Although the DNA yield is not the first concern in PCR-based studies, the acquisition of DNA with high quality and quantity is an essential prerequisite for good reproducibility, which is particular important for samples with low microbial biomass [21,40]. In addition, it has been shown that low concentrations of template DNA in PCR can cause significant variations in profiling microbial communities [45].

Compared to the effect of DNA extraction kits, it seems that the primer choice is a larger source of variation in detection of the eukaryotic microbial communities. Although the two primer sets tested in this study both targeted the V4 region and overlapped by more than 350 bp, the EK primer set severely underestimated the diversity of eukaryotic microorganisms. However, almost 70% of the diversity detected by the TAR primer set are unique OTUs with low abundance. The degeneracy and lower annealing temperature of the TAR primer set may contribute to the high proportion of rare sequences detected, which are known factors that can reduce specificity and increase preferential amplification [46,47]. In addition, it has been pointed that inflated diversity may arise from contamination or sequencing error [11]. Thus, caution should be paid to the ecological implications of low-abundance OTUs. The differences observed between primer sets can also be partially attributed to the relatively longer amplicons (average length: 506 bp vs. 398 bp) obtained using the EK primer set, which appears to be approaching the upper limit of the MiSeq sequencing technology. It has been previously shown that both the PCR kinetics and some polymerases favor short fragments and, thus, divergent and rare taxa can be more readily detected with smaller amplicons [48,49,50]. Another notable difference was the variance of the read abundance between primer sets. The average relative abundance of two OTUs of the same genera (i.e., *Stephanodiscus*, *Aulacoseira*, Figure 6) recovered with two primer sets differed by four orders of magnitude. These results are consistent with studies showing that 16S rRNA primer selection have obvious effects on taxonomic variation [11], which reinforces that a reliable comparison between studies should be performed using the same primer set. But it should be pointed that the striking difference in observed OTU numbers between primers did not result in detection of different range of taxonomic groups at phylum level, suggesting that both primers have a comparable ability in detecting higher-level groups. Meanwhile, a very large fraction of sequences recovered by the TAR primer set cannot be assigned to any known eukaryotic group, which highlights the huge number of undescribed microbial eukaryotes in freshwater sediment habitats and the incompleteness of the reference database.

## 5. Conclusions

Our results highlighted the influence of DNA extraction kit and primer choice on the observed microbial community in freshwater sediment. Overall, the FastDNA SPIN Kit for Soil and DNeasy PowerSoil Kit produced much higher DNA yield with good reproducibility, and observed more eukaryotic OTUs from freshwater sediment samples, while all three kits used in this study exhibited comparable ability in recovering bacterial alpha diversity. The EK primer set is preferred to capture the abundant taxa of protistan community as it shows high level of reproducibility between replicates and more robust in PCR amplification with concise cycling conditions. However, if the diversity of rare species is desired, the TAR primer set in combination with the FastDNA SPIN Kit is suggested to reveal a more complete picture of the eukaryotic microbial community structure.

## Figures and Tables

**Table 1:** Comparison of different DNA extraction kits on DNA yields, purity and number of OTUs *.

Extraction Kit (Replicate)	DNA Yield (ng/μL)	A260/A280	A260/A230	16S		18S (Primer EK)		18S (Primer TAR)	
				Observed OTUs	Number of Reads	Observed OTUs	Number of Reads	Observed OTUs	Number of Reads
PS Kit (1)	12.1	2.12	2.28	3447	53,722	464	54,167	495	56,794
PS Kit (2)	10.7	2.08	0.91	2918	47,465	373	31,698	452	47,406
PS Kit (3)	14.8	1.77	0.85	3569	58,878	414	26,946	723	32,269
PS Kit (4)	9.70	2.44	1.96	3063	37,905	389	34,004	446	68,706
PS Kit (5)	11.00	2.06	1.93	3442	64,948	426	41,928	534	56,171
FS Kit (1)	15.00	1.72	0.03	3082	40,066	332	25,834	659	59,622
FS Kit (2)	14.40	1.95	0.04	3244	33,726	291	26,791	975	63,743
FS Kit (8)	15.00	1.83	0.06	3349	38,358	307	42,675	355	66,368
FS Kit (4)	17.00	1.85	0.04	3785	62,632	342	42,608	904	54,906
FS Kit (5)	25.30	1.53	0.75	3095	36,319	324	33,241	406	30,630
MG Kit (1)	1.80	2.67	1.99	3254	51,613	168	43,146	146	50,094
MG Kit (2)	3.00	2.41	2.55	4072	64,839	160	58,909	92	21,079
MG Kit (3)	1.10	2.08	0.02	4129	67,204	156	62,365	86	32,158
MG Kit (4)	1.20	2.06	1.78	3541	41,225	109	28,986	67	56,659
MG Kit (5)	0.70	1.96	1.98	3950	52,999	115	27,190	108	48,887

* The number of reads and OTUs are obtained after quality filtering and before rarefication.

## Data Availability

Assembled sequences are deposited in the NCBI Sequence Reads Archive under BioProject PRJNA848776.

## Supplementary Materials

The following supporting information can be downloaded at: https://www.mdpi.com/article/10.3390/microorganisms10061213/s1, Figure S1. Relative abundances of bacterial communities at phylum level in samples extracted using the FS, PS and MG kit; Figure S2. Venn diagram showing the number of shared and unique eukaryotic OTUs detected by two primer sets (EK-565F/134 & TAReukV4F/RevR) among the samples extracted using the PS, FS and MG kit; Table S1. Comparison of shared and unique OTUs and their abundance in prokaryotes among three DNA extraction kits; Table S2. Numbers in bold indicate taxa are differentially abundant between kits.
