# Supplementary material for: The Effects of DNA Extraction Kits and Primers on Prokaryotic and Eukaryotic Microbial Community in Freshwater Sediments
Source: Microorganisms. 2022 Jun 14;10(6):1213. doi: 10.3390/microorganisms10061213 (PMC9230960; doi:10.3390/microorganisms10061213)
Supplement: Supplementary file 1 [file microorganisms-10-01213-s001.zip › Supplementary Information.pdf]

Supplementary Information

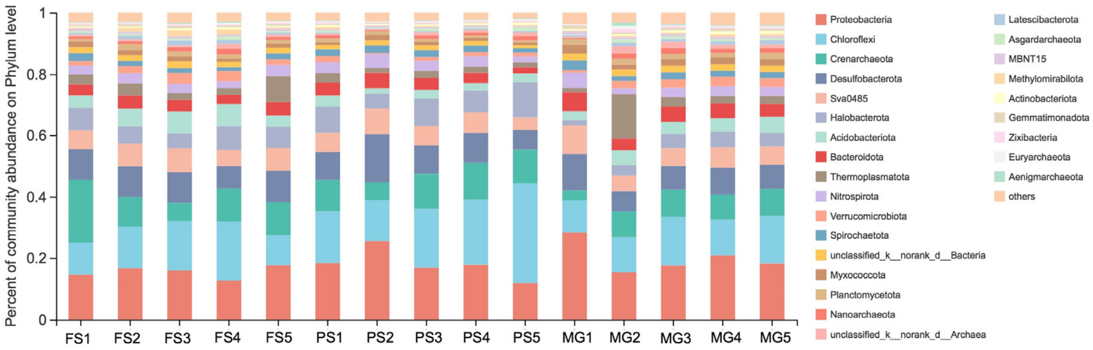

Fig. S1 Relative abundances of bacterial communities at phylum level in samples extracted using the FS, PS and MG kit.

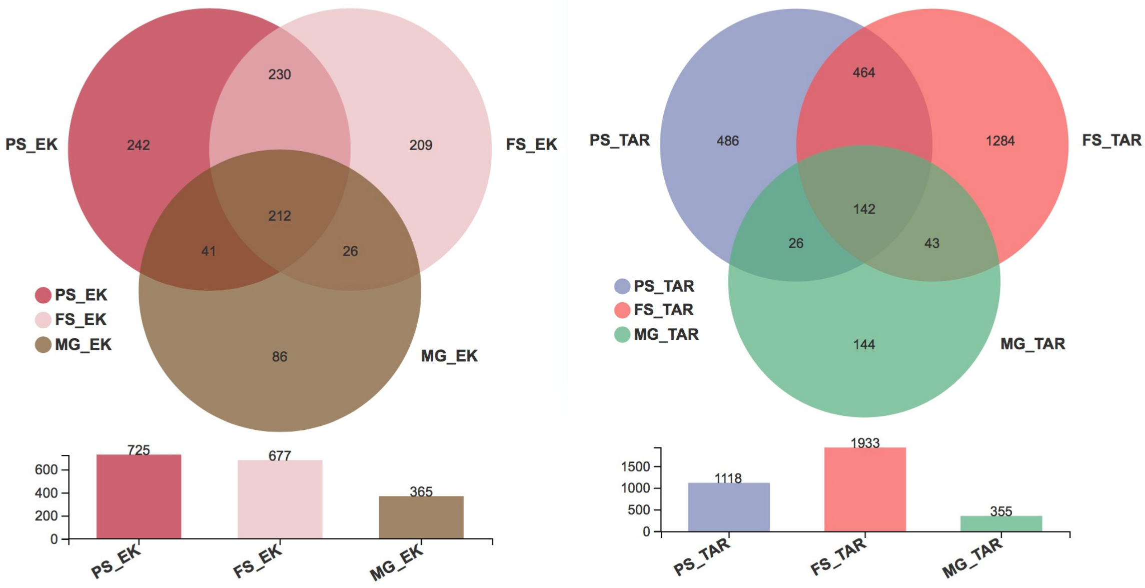

Fig. S2 Venn diagram showing the number of shared and unique eukaryotic OTUs detected by two primer sets (EK-565F/134 & TAReukV4F/RevR) among the samples extracted using the PS, FS and MG kit.

Table S1. Comparison of shared and unique OTUs and their abundance in prokaryotes among three DNA extraction kits.

Shared OTUs										Unique OTUs					
All				PS-FS		FS-MG		PS-MG		PS		FS		MG	
No. of OTUs	RA (%)			No. of OTUs	RA (%)	No. of OTUs	RA (%)	No. of OTUs	RA (%)	No. of OTUs	RA (%)	No. of OTUs	RA (%)	No. of OTUs	RA (%)
	PS	FS	MG												
4037	99.28	99.01	98.32	4189	99.40	4492	99.39	4299	99.17	33	0.053	51	0.074	185	0.61

RA: Relative Abundance
